# 3-Amino­phenyl­boronic acid monohydrate

**DOI:** 10.1107/S1600536810015655

**Published:** 2010-05-08

**Authors:** Araceli Vega, Maria Zarate, Hugo Tlahuext, Herbert Höpfl

**Affiliations:** a Centro de Investigaciones Químicas, Universidad Autónoma del Estado de Morelos, Av. Universidad 1001, Col. Chamilpa, CP 62209, Cuernavaca, Mexico

## Abstract

In the title compound, C_6_H_8_BNO_2_·H_2_O, the almost planar boronic acid mol­ecules (r.m.s. deviation = 0.044 Å) form inversion dimers, linked by pairs of O—H⋯O hydrogen bonds. The water mol­ecules link these dimers into [100] chains by way of O—H⋯O hydrogen bonds, and N—H⋯O links generate (100) sheets.

## Related literature

For background to the synthesis, structures and applications of phenyl­boronic acid derivatives, see: Barba & Betanzos (2007 ▶); Barba *et al.* (2004 ▶, 2006 ▶); Bernstein *et al.* (1995 ▶); Christinat *et al.* (2008 ▶); Dreos *et al.* (2002 ▶); Fujita *et al.* (2008 ▶); Höpfl (2002 ▶); Hall (2005 ▶); Lulinski *et al.* (2007 ▶); Miyaura & Suzuki (1995 ▶); Severin (2009 ▶); Shinkai *et al.* (2001 ▶); Smith *et al.* (2008 ▶); Zhang *et al.* (2007 ▶).
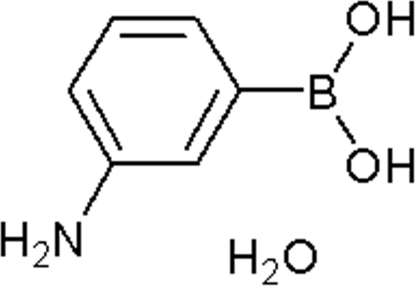


## Experimental

### 

#### Crystal data

C_6_H_8_BNO_2_·H_2_O
*M* *_r_* = 154.96
Monoclinic, 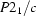

*a* = 7.1211 (8) Å
*b* = 13.8548 (15) Å
*c* = 7.8475 (8) Å
β = 100.663 (2)°
*V* = 760.88 (14) Å^3^
*Z* = 4
Mo *K*α radiation
μ = 0.11 mm^−1^
*T* = 100 K
0.44 × 0.38 × 0.34 mm

#### Data collection

Bruker SMART APEX CCD diffractometer
Absorption correction: multi-scan (*SADABS*; Sheldrick, 1996 ▶) *T*_min_ = 0.89, *T*_max_ = 1.00
7077 measured reflections
1341 independent reflections
1258 reflections with *I* > 2σ(*I*)
*R*_int_ = 0.022

#### Refinement

*R*[*F* ^2^ > 2σ(*F* ^2^)] = 0.032
*wR*(*F* ^2^) = 0.088
*S* = 1.03
1341 reflections
124 parameters
6 restraints
H atoms treated by a mixture of independent and constrained refinement
Δρ_max_ = 0.29 e Å^−3^
Δρ_min_ = −0.17 e Å^−3^

### 

Data collection: *SMART* (Bruker, 2000 ▶); cell refinement: *SAINT-Plus-NT* (Bruker, 2001 ▶); data reduction: *SAINT-Plus-NT*; program(s) used to solve structure: *SHELXTL-NT* (Sheldrick, 2008 ▶); program(s) used to refine structure: *SHELXTL-NT*; molecular graphics: *SHELXTL-NT*; software used to prepare material for publication: *PLATON* (Spek, 2009 ▶) and *publCIF* (Westrip, 2010 ▶).

## Supplementary Material

Crystal structure: contains datablocks I, global. DOI: 10.1107/S1600536810015655/hb5409sup1.cif

Structure factors: contains datablocks I. DOI: 10.1107/S1600536810015655/hb5409Isup2.hkl

Additional supplementary materials:  crystallographic information; 3D view; checkCIF report

## Figures and Tables

**Table 1:** Hydrogen-bond geometry (Å, °)

*D*—H⋯*A*	*D*—H	H⋯*A*	*D*⋯*A*	*D*—H⋯*A*
O1—H1′⋯O2^i^	0.84 (1)	1.92 (1)	2.7583 (13)	174 (2)
N1—H1*A*⋯O31^ii^	0.86 (1)	2.21 (1)	3.0661 (15)	177 (1)
N1—H1*B*⋯O1^iii^	0.86 (1)	2.43 (1)	3.1854 (15)	147 (1)
O2—H2′⋯O31	0.84 (1)	1.91 (1)	2.7159 (13)	161 (2)
O31—H31*A*⋯N1^iv^	0.84 (1)	2.07 (1)	2.9040 (15)	173 (2)
O31—H31*B*⋯O1^v^	0.84 (1)	2.05 (1)	2.8810 (13)	170 (2)

Symmetry codes: (i) 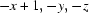; (ii) 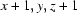; (iii) 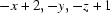; (iv) 
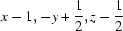
; (v) 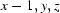.

**Fig. 1.**
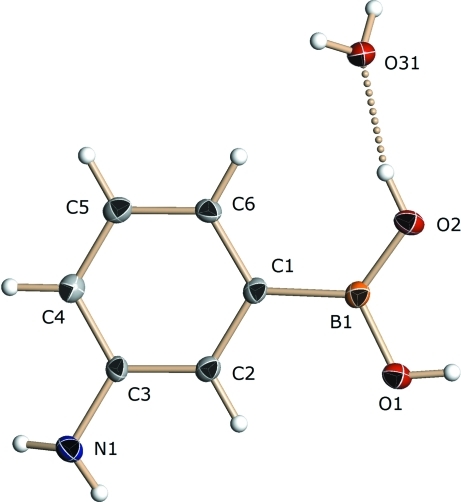
Perspective view of (I) with displacement ellipsoids drawn at the 50% probability level.

**Fig. 2.**
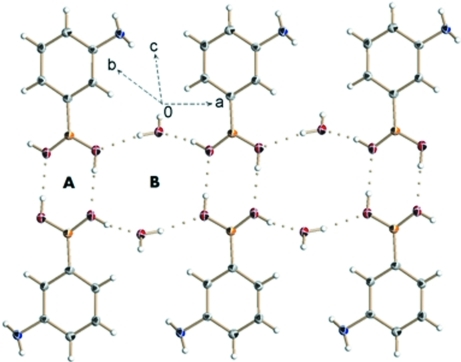
In the crystal structure of (I) homodimeric boronic acid motifs A and water-expanded motifs B are linked to 1D hydrogen-bonded chains.

## supplementary crystallographic information

### Comment

Substituted phenylboronic acid derivatives have been prepared mainly for applications in organic synthesis (Miyaura & Suzuki, 1995; Hall, 2005) and for molecular recognition of biochemically active molecules (Shinkai *et al.*, 2001). More recently, such boronic acid derivatives have attracted attention also as building blocks for the self-assembly of macrocyclic and polymeric assemblies. For this purpose, the boronic acid is generally converted to an ester (boronate) via condensation with an aliphatic or aromatic diol, which is then assembled to a macromolecular structure via reaction of the additional functional group attached to the *B*-phenyl ring (Höpfl, 2002; Fujita *et al.*, 2008; Severin, 2009). In this context, 3-aminophenylboronic acid has been employed for the generation of macrocycles and cages (Dreos *et al.*, 2002; Barba *et al.*, 2004 and 2006; Barba & Betanzos, 2007; Christinat *et al.*, 2008).

We report herein on the molecular and crystal structure of 3-aminophenylboronic acid monohydrate (I).

The asymmetric unit of I contains one 3-aminophenylboronic acid and one water molecule (Figure 1). The boronic acid molecules are associated through the well-known -B(OH)_2_···(HO)_2_B-synthon (motif A) with the graph set *R*_2_^2^(8) (Bernstein *et al.*, 1995), in which each B(OH)_2_ group has *syn*-*anti *conformation (with respect to the H atoms), thus allowing for the formation of additional hydrogen bonds with the water molecules included in the crystal lattice. These (B)O—H···O_w_ hydrogen bonds give rise to a cyclic water-expanded motif B [graph set *R*_6_^6^(12)] of the boronic acid homodimer, thus generating a 1D chain along axis *a* (Figure 2). The (OH)_6_ ring has chair-conformation and has been observed previously in the crystal structures of 3,5-dibromo-2-formylphenylboronic acid monohydrate (Lulinski *et al.*, 2007), 5-quinolineboronic acid monohydrate (Zhang *et al.*, 2007) and 2,6-dichloro-3-pyridylboronic acid hemihydrate (Smith *et al.*, 2008). The 1D chains are interconnected through O_w_—H···N, N—H···O_w_ and N—H···O(B) hydrogen bonds to give an overall 3D hydrogen bonded network (Table 1).

### Experimental

3-Aminophenylboronic acid monohydrate is a commercially available product that has been crystallized from a solvent mixture of benzene, methanol and water to generate colourless blocks of (I); M.p. 368 K.

### Refinement

H atoms were positioned geometrically and constrained using the riding-model approximation [*C*-H_aryl _= 0.93 Å, *U*_iso_(H_aryl_)= 1.2 *U*_eq_(C)]. Hydrogen atoms bonded to O (H1', H2', H31A and H31B) and N (H1A and H1B) were located in difference Fourier maps. The coordinates of the O—H and N—H hydrogen atoms were refined with distance restraints: O—H = 0.84±0.01 Å, N—H = 0.86 Å ±0.01 and [*U*_iso_(H) = 1.5 *U*_eq_(O,*N*)].

### Crystal data

**Table:**

C_6_H_8_BNO_2_·H_2_O	*F*(000) = 328
*M**_r_* = 154.96	*D*_x_ = 1.353 Mg m^−^^3^
Monoclinic, *P*2_1_/*c*	Melting point: 368 K
Hall symbol: -P 2ybc	Mo *K*α radiation, λ = 0.71073 Å
*a* = 7.1211 (8) Å	Cell parameters from 4929 reflections
*b* = 13.8548 (15) Å	θ = 2.9–28.3°
*c* = 7.8475 (8) Å	µ = 0.11 mm^−^^1^
β = 100.663 (2)°	*T* = 100 K
*V* = 760.88 (14) Å^3^	Block, colourless
*Z* = 4	0.44 × 0.38 × 0.34 mm

### Data collection

**Table:**

Bruker SMART APEX CCD diffractometer	1341 independent reflections
Radiation source: fine-focus sealed tube	1258 reflections with *I* > 2σ(*I*)
graphite	*R*_int_ = 0.022
Detector resolution: 8.3 pixels mm^-1^	θ_max_ = 25.0°, θ_min_ = 2.9°
phi and ω scans	*h* = −8→8
Absorption correction: multi-scan (*SADABS*; Sheldrick, 1996)	*k* = −16→16
*T*_min_ = 0.89, *T*_max_ = 1.00	*l* = −9→9
7077 measured reflections	

### Refinement

**Table:**

Refinement on *F*^2^	Primary atom site location: structure-invariant direct methods
Least-squares matrix: full	Secondary atom site location: difference Fourier map
*R*[*F*^2^ > 2σ(*F*^2^)] = 0.032	Hydrogen site location: inferred from neighbouring sites
*wR*(*F*^2^) = 0.088	H atoms treated by a mixture of independent and constrained refinement
*S* = 1.03	*w* = 1/[σ^2^(*F*_o_^2^) + (0.0487*P*)^2^ + 0.3165*P*] where *P* = (*F*_o_^2^ + 2*F*_c_^2^)/3
1341 reflections	(Δ/σ)_max_ < 0.001
124 parameters	Δρ_max_ = 0.29 e Å^−^^3^
6 restraints	Δρ_min_ = −0.17 e Å^−^^3^

### Special details

**Table:**

Geometry. All esds (except the esd in the dihedral angle between two l.s. planes) are estimated using the full covariance matrix. The cell esds are taken into account individually in the estimation of esds in distances, angles and torsion angles; correlations between esds in cell parameters are only used when they are defined by crystal symmetry. An approximate (isotropic) treatment of cell esds is used for estimating esds involving l.s. planes.
Refinement. Refinement of *F*^2^ against ALL reflections. The weighted *R*-factor wR and goodness of fit *S* are based on *F*^2^, conventional *R*-factors *R* are based on *F*, with *F* set to zero for negative *F*^2^. The threshold expression of *F*^2^ > σ(*F*^2^) is used only for calculating *R*-factors(gt) etc. and is not relevant to the choice of reflections for refinement. *R*-factors based on *F*^2^ are statistically about twice as large as those based on *F*, and *R*- factors based on ALL data will be even larger.

### Fractional atomic coordinates and isotropic or equivalent isotropic displacement parameters (Å^2^)

**Table:**

	*x*	*y*	*z*	*U*_iso_*/*U*_eq_	
B1	0.5617 (2)	0.06346 (10)	0.24383 (18)	0.0160 (3)	
N1	1.02034 (15)	0.15487 (8)	0.78329 (14)	0.0186 (3)	
H1A	1.030 (2)	0.1547 (11)	0.8942 (3)	0.022 (4)*	
H1B	1.0986 (18)	0.1138 (9)	0.753 (2)	0.029 (4)*	
O1	0.71516 (12)	0.03223 (7)	0.17614 (11)	0.0180 (2)	
H1'	0.682 (3)	0.0084 (12)	0.0767 (10)	0.038 (5)*	
O2	0.38624 (12)	0.05950 (6)	0.13944 (11)	0.0179 (2)	
H2'	0.2917 (15)	0.0828 (12)	0.175 (2)	0.036 (5)*	
C1	0.60035 (17)	0.10356 (8)	0.43486 (16)	0.0151 (3)	
C2	0.78720 (17)	0.10598 (8)	0.52950 (16)	0.0158 (3)	
H2	0.8881	0.0809	0.4786	0.019*	
C3	0.82923 (17)	0.14429 (8)	0.69650 (16)	0.0151 (3)	
C4	0.68011 (18)	0.17961 (9)	0.77225 (16)	0.0171 (3)	
H4	0.7062	0.2051	0.8866	0.021*	
C5	0.49436 (18)	0.17741 (9)	0.68042 (16)	0.0181 (3)	
H5	0.3935	0.2018	0.7322	0.022*	
C6	0.45380 (17)	0.13999 (9)	0.51337 (16)	0.0163 (3)	
H6	0.3257	0.1391	0.4519	0.020*	
O31	0.05437 (12)	0.14588 (7)	0.17821 (12)	0.0199 (2)	
H31A	0.048 (3)	0.2022 (5)	0.217 (2)	0.041 (5)*	
H31B	−0.0485 (14)	0.1179 (12)	0.186 (2)	0.040 (5)*	

### Atomic displacement parameters (Å^2^)

**Table:**

	*U*^11^	*U*^22^	*U*^33^	*U*^12^	*U*^13^	*U*^23^
B1	0.0179 (7)	0.0120 (7)	0.0181 (7)	−0.0012 (5)	0.0037 (6)	0.0013 (5)
N1	0.0161 (6)	0.0235 (6)	0.0156 (6)	0.0018 (4)	0.0016 (4)	−0.0009 (4)
O1	0.0155 (5)	0.0228 (5)	0.0152 (5)	0.0002 (4)	0.0017 (3)	−0.0049 (4)
O2	0.0143 (5)	0.0221 (5)	0.0172 (5)	0.0019 (4)	0.0026 (4)	−0.0047 (4)
C1	0.0174 (6)	0.0110 (6)	0.0171 (6)	−0.0019 (5)	0.0034 (5)	0.0016 (5)
C2	0.0167 (6)	0.0137 (6)	0.0179 (6)	0.0010 (5)	0.0058 (5)	0.0014 (5)
C3	0.0164 (6)	0.0126 (6)	0.0158 (6)	−0.0008 (5)	0.0019 (5)	0.0030 (5)
C4	0.0206 (7)	0.0153 (6)	0.0156 (6)	−0.0012 (5)	0.0038 (5)	−0.0013 (5)
C5	0.0177 (6)	0.0159 (6)	0.0219 (7)	0.0011 (5)	0.0072 (5)	−0.0007 (5)
C6	0.0135 (6)	0.0157 (6)	0.0190 (6)	−0.0015 (5)	0.0013 (5)	0.0004 (5)
O31	0.0154 (5)	0.0228 (5)	0.0219 (5)	0.0007 (4)	0.0045 (4)	−0.0031 (4)

### Geometric parameters (Å, °)

**Table:**

B1—O2	1.3623 (17)	C2—C3	1.3941 (18)
B1—O1	1.3707 (17)	C2—H2	0.9500
B1—C1	1.5745 (18)	C3—C4	1.3980 (18)
N1—C3	1.4122 (16)	C4—C5	1.3846 (18)
N1—H1A	0.860 (3)	C4—H4	0.9500
N1—H1B	0.860 (13)	C5—C6	1.3894 (18)
O1—H1'	0.840 (10)	C5—H5	0.9500
O2—H2'	0.840 (13)	C6—H6	0.9500
C1—C2	1.3991 (17)	O31—H31A	0.842 (9)
C1—C6	1.4005 (18)	O31—H31B	0.841 (12)
			
O2—B1—O1	117.55 (11)	C2—C3—C4	119.02 (11)
O2—B1—C1	124.48 (11)	C2—C3—N1	120.86 (11)
O1—B1—C1	117.95 (11)	C4—C3—N1	119.91 (11)
C3—N1—H1A	112.3 (11)	C5—C4—C3	119.91 (11)
C3—N1—H1B	114.4 (11)	C5—C4—H4	120.0
H1A—N1—H1B	109.9 (15)	C3—C4—H4	120.0
B1—O1—H1'	112.2 (13)	C4—C5—C6	120.73 (11)
B1—O2—H2'	119.1 (12)	C4—C5—H5	119.6
C2—C1—C6	118.08 (11)	C6—C5—H5	119.6
C2—C1—B1	119.71 (11)	C5—C6—C1	120.52 (11)
C6—C1—B1	122.19 (11)	C5—C6—H6	119.7
C3—C2—C1	121.72 (11)	C1—C6—H6	119.7
C3—C2—H2	119.1	H31A—O31—H31B	107.4 (18)
C1—C2—H2	119.1		
			
O2—B1—C1—C2	−178.77 (11)	C1—C2—C3—N1	−173.67 (11)
O1—B1—C1—C2	−0.23 (17)	C2—C3—C4—C5	−0.90 (18)
O2—B1—C1—C6	−0.64 (19)	N1—C3—C4—C5	173.96 (11)
O1—B1—C1—C6	177.90 (11)	C3—C4—C5—C6	0.28 (18)
C6—C1—C2—C3	−0.73 (18)	C4—C5—C6—C1	0.13 (18)
B1—C1—C2—C3	177.47 (11)	C2—C1—C6—C5	0.08 (18)
C1—C2—C3—C4	1.14 (18)	B1—C1—C6—C5	−178.07 (11)

### Hydrogen-bond geometry (Å, °)

**Table:**

*D*—H···*A*	*D*—H	H···*A*	*D*···*A*	*D*—H···*A*
O1—H1'···O2^i^	0.84 (1)	1.92 (1)	2.7583 (13)	174 (2)
N1—H1A···O31^ii^	0.86 (1)	2.21 (1)	3.0661 (15)	177 (1)
N1—H1B···O1^iii^	0.86 (1)	2.43 (1)	3.1854 (15)	147 (1)
O2—H2'···O31	0.84 (1)	1.91 (1)	2.7159 (13)	161 (2)
O31—H31A···N1^iv^	0.84 (1)	2.07 (1)	2.9040 (15)	173 (2)
O31—H31B···O1^v^	0.84 (1)	2.05 (1)	2.8810 (13)	170 (2)

Symmetry codes: (i) −*x*+1, −*y*, −*z*; (ii) *x*+1, *y*, *z*+1; (iii) −*x*+2, −*y*, −*z*+1; (iv) *x*−1, −*y*+1/2, *z*−1/2; (v) *x*−1, *y*, *z*.
